# Bacteriology

**Published:** 1905-07-01

**Authors:** 


### BACTERIOLOGY.

**Diphtheria.**—Petrie[1] has conducted a valuable research into the question of the identity of the Hoffmann and diphtheria bacillus. He notes that Spronck found that the local reaction produced by subcutaneous injection of pseudo-diphtheria bacilli was not influenced by the subsequent injection of diphtheria antitoxin; Glücksmann that immunisation by Hoffmann's bacillus did not confer immunity against diphtheria bacilli subsequently injected; Lubowski obtained negative agglutination results with true diphtheria bacilli; and that Cobbett was unable to confirm the observations made by Salter and others—namely, that in the filtrates of the Hoffmann bacillus substances exist which are identical with the toxoids present in diphtheria toxin. Petrie sought to prove the presence or absence of such toxoids by (1) adding quantities of pseudo-diphtheria filtrates to toxin antitoxin mixture, and (2) by immunising horses with large quantities of the filtrates and examining the serum afterwards for antitoxin. His Hoffmann cultures were marked by the following characteristics:—They stained by Gram's but not by Neisser's method. They gave an alkaline reaction in glucose broth. Cultures stained uniformly, and when old showed few involution forms. Broth cultures inoculated into guinea pigs gave rise to no symptoms. His results may be given in his own words. "No substances capable of neutralising diphtheria antitoxin are present in filtrates of pseudo-diphtheria bacilli. The results of the immunisation of horses with large quantities of the filtrates makes it apparent that they do not contain substances capable of stimulating the production of an antitoxin to diphtheria toxin." These results diminish the probability of there being any relationship between the Hoffmann and Klebs Lœffler bacillus, and demonstrate that when the two are associated in any pathological condition no toxoids are elaborated by the Hoffmann variety which would interfere with the action of antitoxin administered therapeutically. Rolleston [2] points out that the nontherapeutical sequelæ of diphtheria antitoxin are due not to any specific toxic bodies but to the pre ence of unknown bodies in normal horse serum. He has noted as of favourable prognosis a drowsiness setting in soon after the injection. Over 70 per cent. of cases injected with serum subsequently develop rashes, the frequency of the rash bearing in direct relation to the size of the dose. Three types of rashes are seen, scarlatiniform, urticarial, and erythematous. The scarlatiniform rash occurs early in the disease and it is doubtful whether it is directly attributable to the serum. Urticarial rashes appear about the seventh or eighth day after injection, and may be accompanied by a rise of temperature. The rash generally subsides in two or three days, but successive crops may appear for ten days. Circinate erythema generally follows urticaria, is found from the tenth to the eighteenth day, and is accompanied by fever, joint pains, and adenitis. Hyperidrosis is a phenomenon following the use of anti-diphtheritic serum. Albuminuria is certainly caused by the injections, but is of no serious importance. The more marked the antitoxin reaction the better is the prognosis, and the more marked the secondary rash and its concomitant symptoms the less chance is there of severe subsequent paralysis. Diphtheria antitoxin has also been used by Waitzfelder [3] for the cure of epidemic cerebro-spinal meningitis. Of seventeen cases so treated, three died, five recovered, and nine remained under treatment, the majority of these being convalescent. The dose varied from 5,000 to 10,000 units, given on alternate days, and there followed a marked relief of the general symptoms. The important subject of the early diagnosis of diphtheria from the examination of stained films direct from the exudate has been further investigated by Higley.[4] Two staining solutions are employed—(1) Five minims of Kühne's methylene carbolic blue in seven ccms. of distilled water; (2) Ten minims of carbol fuchsin in seven ccms. of tap water. The films, fixed by passing through a flame, are stained for five minutes in No. 1 solution, washed and dried, and then stained for one minute in No. 2 solution. The diphtheria bacillus is stained a dark red or violet, showing polar dots and staining unevenly. The whole process, it is claimed, need not occupy more than fifteen minutes.

**The Bacteria of the Stomach.**—Palier [5] has made a bacteriological examination of the gastric juice in various pathological conditions. In carcinoma ventriculi, a prominent feature was the absence of fungi and the presence of staphylococci. These cocci may account for the pus cells which are, according to this writer, constantly present in the stomach contents in malignant disease. Yeast cells he found to be present in gastric contents which did not contain hydrochloric acid. In all the cases of carcinoma Palier found the Boas-Oppler bacillus. He describes it as 4$\mu$ in length, non-motile, anærobic, and non-sporing. The bacilli form angles with one another (vibrio geniculatus), they stain readily with Gram's solution, and all the usual aniline dyes. Whilst it may be concerned in the formation of lactic acid it is not essentially a lactic acid-forming organism, but in the opinion of the writer a bacillus of putrefaction growing readily in any medium deficient in hydrochloric acid. The absence of fungi in the stomach contents is probably due to the presence of the antagonistic staphylococci. In cases of hypochlorhydria only a few colonies of staphylococci were found in one case, and the distinguishing feature was a free and profuse and varied growth of fungi in all the cases. Cases of hyperchlorhydria, with old ulceration of the stomach, all yielded growth both of yeast and fungi (Schemmelpilze), which fungi were, in the writer's opinion, not true organisms but filamentous outgrowths from the yeast cells. In hyperchlorhydria, also, sarcinæ were found, and a small unnamed bacillus giving off an ammoniacal odour. In subacidity of the stomach there were found yeast, mycelia, and vibrio geniculatus.

[1] Jour. of Hygiene, April 1905. [2] Prac., May 1905. [3] Med. Rec., March 11, 1905. [4] Ibid., April 1, 1905. [5] Ibid., Nov. 19, 1904.

